# Determination and stability of N-terminal pro-brain natriuretic peptide in saliva samples for monitoring heart failure

**DOI:** 10.1038/s41598-021-92488-2

**Published:** 2021-06-22

**Authors:** Francesca G. Bellagambi, Christina Petersen, Pietro Salvo, Silvia Ghimenti, Maria Franzini, Denise Biagini, Marie Hangouët, Maria Giovanna Trivella, Fabio Di Francesco, Aldo Paolicchi, Abdelhamid Errachid, Roger Fuoco, Tommaso Lomonaco

**Affiliations:** 1 Department of Chemistry and Industrial Chemistry, University of Pisa, 56124 Pisa, Italy; 2 Institute of Analytical Sciences (ISA) – UMR 5280, University Claude Bernard Lyon 1, 69100 Lyon, France; 3 Cardiology Division, Fondazione Toscana Gabriele Monasterio, 56124 Pisa, Italy; 4 Institute of Clinical Physiology, National Research Council, 56124 Pisa, Italy; 5 Department of Translational Research on New Technologies in Medicine and Surgery, University of Pisa, 56126 Pisa, Italy; 6 Institute of Analytical Sciences (ISA), UMR 5280, French National Center for Scientific Research (CNRS), 69100 Lyon, France

**Keywords:** Biomarkers, Cardiology, Chemistry

## Abstract

Heart failure (HF) is the main cause of mortality worldwide, particularly in the elderly. N-terminal pro-brain natriuretic peptide (NT-proBNP) is the gold standard biomarker for HF diagnosis and therapy monitoring. It is determined in blood samples by the immunochemical methods generally adopted by most laboratories. Saliva analysis is a powerful tool for clinical applications, mainly due to its non-invasive and less risky sampling. This study describes a validated analytical procedure for NT-proBNP determination in saliva samples using a commercial Enzyme-Linked Immuno-Sorbent Assay. Linearity, matrix effect, sensitivity, recovery and assay-precision were evaluated. The analytical approach showed a linear behaviour of the signal throughout the concentrations tested, with a minimum detectable dose of 1 pg/mL, a satisfactory NT-proBNP recovery (95–110%), and acceptable precision (coefficient of variation ≤ 10%). Short-term (3 weeks) and long-term (5 months) stability of NT-proBNP in saliva samples under the storage conditions most frequently used in clinical laboratories (4, − 20, and − 80 °C) was also investigated and showed that the optimal storage conditions were at − 20 °C for up to 2.5 months. Finally, the method was tested for the determination of NT-proBNP in saliva samples collected from ten hospitalized acute HF patients. Preliminary results indicate a decrease in NT-proBNP in saliva from admission to discharge, thus suggesting that this procedure is an effective saliva-based point-of-care device for HF monitoring.

## Introduction

Heart failure (HF) is a pathophysiological condition that causes an inadequate blood supply to all the organs and apparatus. This is particularly due to the impairment of the heart’s capacity to pump out blood or to fill one or both ventricles. HF is an increasingly common chronic cardiovascular disease and, according to the World Health Organization, the main cause of mortality and major morbidity worldwide, particularly in the elderly^1, 2^.

Approximately thirty million people worldwide are affected by HF^3^, however this does not include undiagnosed or misdiagnosed cases^4^. HF causes high mortality rates in elderly patients and is a heavy economic burden on national health services^5–7^.

The diagnosis of HF in some patients is made more challenging due to nonspecific signs and symptoms^8, 9^, with possible risks to the patient's health and additional costs for the health services. Early diagnosis and therapy monitoring should therefore be improved to minimize the impact of HF on the population^10^.

Biomarkers are commonly described as biochemical compounds that provide information on normal biological processes, pathogenic processes or responses to an exposure or intervention^11^. Several biomarkers have been considered for HF management^12–16^. Natriuretic peptides (NP), such as Brain Natriuretic Peptide (BNP) and the N-terminal proBNP (NT-proBNP), have been identified as gold standard biomarkers of HF by both European and American guidelines^17, 18^. Increased plasma levels of circulating NP in patients with congestive HF are directly related to the severity of congestive heart failure, as classified by the New York Heart Association criteria^19^.

Measuring the plasma or the serum concentrations of both BNP and NT-proBNP is therefore currently recommended to support the diagnosis of HF^13, 20^. NT-proBNP has a very high prognostic power due to its correlation with the mortality, morbidity, and hospitalization rate of HF patients^18, 21^. In addition, NT-proBNP shows additional advantages over BNP in diagnosing and assessing the severity of HF, such as a higher circulating concentration and longer stability^22^.

Blood is generally regarded as the best body fluid to evaluate systemic processes through the determination of biomarkers, in which NT-proBNP is the gold standard biomarker for HF diagnosis and monitoring^16, 23^. However, blood sampling can be stressful for patients due to its potential risks, such as transient discomfort, bruising, infection at the venipuncture site, and anemia^24^. Moreover, blood sample manipulation requires particular treatments, i.e. both in terms of sample analysis and disposal.

Saliva analysis is an increasingly common alternative method to blood testing. Saliva (i.e. whole saliva) is an “ultra-filtrate” of blood and has gained importance as a potential source of clinical information because it reflects biological activity as well as a healthy or pathological status. Compared with blood, saliva samples can be easily and unobtrusively collected, even from critical subjects (e.g. children, the elderly, and the disabled). Non-invasive saliva sampling is suitable for the screening of a large population, and decreases psychological stress (especially if repeated sampling is needed), and health risks for patients and healthcare professionals^25–31^. In addition, salivary diagnostics is being exploited in Lab-on-Chip (LoC) and Point-of-Care (PoC) devices^32, 33^.

However, there are currently no robust information on the salivary levels of NT-proBNP as HF biomarkers or reliable methods for its determination in saliva.

In fact, BNP and NT-proBNP are usually quantified in blood or plasma by immunoassays, such as the Enzyme-Linked Immuno-Sorbent Assay (ELISA)^34–37^, electrochemiluminescence immunoassay (ECLIA) and radioimmunoassay (RIA)^38^, immunoradiometric assay (IRMA)^39^, and fluorescent immunochromatographic assay (FICA)^40^. In addition, affinity chromatography and chromatography coupled to tandem mass spectrometry methods^41, 42^ can be used for NP determination in blood. ELISAs are the most common procedure for HF biomarker quantification, however commercially available immunoassay kits are generally intended to analyze cell culture supernates, serum, EDTA plasma, heparin plasma, and citrate plasma.

One of the most widely used immuneassays for NT-proBNP quantification in plasma and serum sample is the Elecsys NT-proBNP II assay from Roche^43–46^. This is an automated electrochemiluminescent immunoassay for NT-proBNP quantification in a concentration range of 10–35,000 pg/mL, with a detection limit (LoD) of 10 pg/mL and limit of quantification (LoQ) of 50 pg/mL. In 2012, Foo et al.^47^ used the Elecsys NT-proBNP II assay to validate their immunoassay in order to quantify NT-proBNP in saliva. Foo et al. used the NT-proBNP AlphaLISA kit from Perkin Elmer. This kit is sold for the quantitative determination of NT-proBNP in buffer, plasma, and serum, in a concentration range of 3.9–100,000 pg/mL, with a LoD of 3.9 pg/mL and a LOQ of 10.2 pg/mL. Foo et al. also investigated the assay performance characteristics of the NT-proBNP AlphaLISA immunoassay for saliva analysis in terms of recovery, intra-and inter-assay coefficient of variation, and LOD. Foo et al. found a % recovery of 85%, an intra-assay variation of 7.17% (± 0.75%), an inter-assay variation of 4.46% (± 0.59%), and an LoD of 16 pg/mL. However, they did not validate the Elecsys NT-proBNP II assay for saliva analysis specifically, and they did not investigate the NT-proBNP stability in saliva in relation to different storage conditions.

In this study, we report the validation of an analytical method to quantify NT-proBNP in saliva samples based on a commercial ELISA kit designed for the quantitative determination of NT-proBNP in human serum/plasma. To the best of our knowledge, this is the first time that the stability of NT-proBNP has been investigated in saliva samples stored for up to 3 weeks at 4 °C (short-term stability study) and up to 5 months at both − 20 and − 80 °C (long-term stability study). The effect of the thaw/freezing cycle was also evaluated.

Finally, we used our method to determine NT-proBNP in saliva samples collected from ten acute HF patients to highlight the potential difference in saliva NT-proBNP levels between hospital admission and discharge. The aim of our paper is not to correlate NT-proBNP levels in plasma and saliva, but to test and validate an analytical approach based on a plasma/serum NT-proBNP ELISA kit for its potential use in a saliva-based PoC.

## Results

### Assay validation for saliva analysis

In this study, an ELISA kit originally commercialized to determine NT-proBNP in human serum and EDTA plasma samples was validated for saliva samples. Linearity, matrix effect, sensitivity, recovery, intra- and inter-assay precision of the ELISA kit were evaluated using quality control samples (QCSs) prepared by spiking aliquots of pooled saliva samples (PSSs), collected from healthy volunteers, with a known amount of analyte.

**Figure 1:**
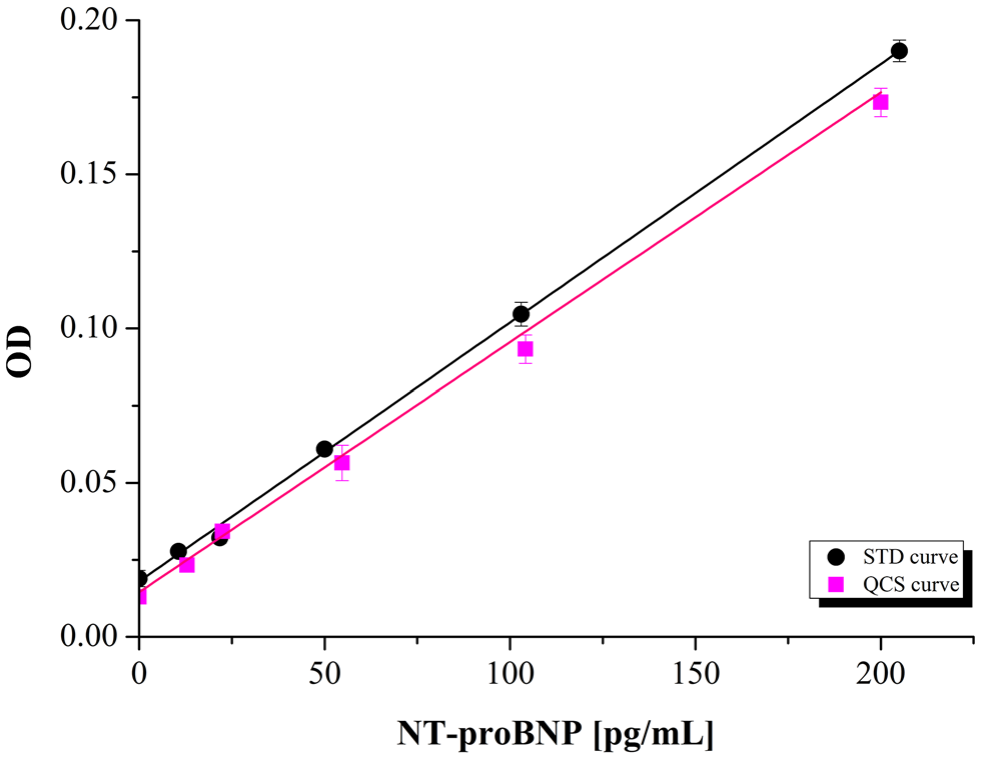
Six-point calibration curves (y = mx + q) obtained from STDs (black) and QCSs (dark pink), and described by y = 0.0008x + 0.01884 (with R^2^ = 0.9996) and y = 0.0008x + 0.01438 (with R^2^ = 0.9987) for STDs and QCSs, respectively.

The assay response was evaluated following the procedure provided by the ELISA kit manufacturer. Method linearity was tested by analyzing both standard solutions (STDs) and QCSs at six concentration levels in the range of 1 – 200 pg/mL, which were selected according to the NT-proBNP salivary levels previously reported by Foo et al.^47^. In this range and for eight calibration curves, the optical density (OD) of NT-proBNP linearly increased with the concentration of both STDs and QCSs (R^2^ ≥ 0.9996 ± 0.0030 and 0.9987 ± 0.0020, respectively), as shown in Fig. 1. Calibration curves (y = mx + q) resulted in y = 0.0008x + 0.01884 and y = 0.0008x + 0.01438 for STDs and QCSs, respectively. A matrix effect was excluded by comparing, at a confidence level of 95%, the slopes of the calibration curves, obtained after subtracting blanks. The two-tailed *p* value (0.49) confirmed the null hypothesis that the slopes were statistically identical.

The minimum detectable dose (MDD) resulted in 1 pg/mL (SD = 1 pg/mL).

The intra-assay precision was evaluated by analyzing five QCSs of a known concentration, ten times each on the same plate, whereas the inter-assay precision was determined by testing the same samples in ten separate assays. Both parameters were expressed as a coefficient of variation (CV%) and were lower than 10% at each concentration level tested. Analyte recovery, determined from ten replicates of each of the five QCSs, ranged from 95 to 110%, with CV% lower than 10% for each concentration tested.

The SOS device did not release any interferents and allowed for a recovery of NT-proBNP equal to 100% (SD = 2%, CV% = 10%) regardless of the concentration level tested (10, 50, and 100 pg/mL).

### Sample stability study

A short-term (T_S_) stability study and a long-term (T_L_) stability study were carried out to evaluate NT-proBNP stability in saliva samples. The T_S_ study investigated analyte stability at 4 °C for up to 3 weeks (T_0_: collection day; T_1S_: T_0_ + 1 week; T_2S_: T_0_ + 2 weeks; T_3S_: T_0_ + 3 weeks), whereas the T_L_ study investigated analyte stability at − 20 and − 80 °C for up to 5 months (T_0_: collection day; T_1L_: T_0_ + 1 month; T_2L_: T_0_ + 2.5 months; T_3L_: T_0_ + 5 months). T_0_ was the same day for both T_S_ and T_L_ studies. More specifically, aliquots of PSS collected and prepared at T_0_ were spiked with 10, 50, and 100 pg/mL of analyte (namely CL_1_, CL_2_, and CL_3_ samples) and immediately analyzed for use as a reference value for both T_S_ and T_L_ studies. The effect of the thaw/freezing cycle was also evaluated at T_1L_.

Table 1 shows the results of the stability studies, which highlighted that NT-proBNP was not stable after 1 week at 4 °C. In such conditions, the concentration of NT-proBNP was lower than the MDD level at both 10 and 50 pg/mL, whereas the concentration of the target analyte measured in the sample containing 100 pg/mL was 13 pg/mL (CV% = 2%). Given these results, the stability of NT-proBNP at 4 °C was not studied for longer storage times. After 1 month (T_1L_), compared with NT-proBNP measured at T_0_, the mean recovery on the three concentration levels was 96% (CV% = 15%) and 87% (CV% = 13%) for samples stored at − 20 and − 80 °C, respectively. Satisfying results were also observed after 2.5 months (T_2L_), with a mean recovery of of 80% and a stable CV = 15% at − 20 °C, whereas a mean recovery of 91% was determined for samples stored at − 80 °C with a CV = 30%. After 5 months (T_3L_) recovery. No statistically significant changes (*p* value > 0.05) in the salivary level of NT-proBNP were observed after two consecutive freeze/thaw cycles.

**Table 1:** Results on stability over time of NT-proBNP in different storage conditions*,* including the mean %recovery. The short-term stability (T_S_) study investigated analyte stability at 4 °C for up to 3 weeks (T_0_: collection day; T_1S_: T_0_ + 1 week; T_2S_: T_0_ + 2 weeks; T_3S_: T_0_ + 3 weeks). The long-term stability (T_L_) study investigated analyte stability at − 20 and − 80 °C for up to 5 months (T_0_: collection day; T_1L_: T_0_ + 1 month; T_2L_: T_0_ + 2.5 months; T_3L_: T_0_ + 5 months). An initial set of samples was analyzed immediately after the collection (T_0_) to obtain the reference values, thus NT-proBNP values measured at T_0_ in the table refer to this sample set. MDD was 1 pg/mL (SD = 1 pg/mL). Each experiment was performed in triplicate.

Short-term stability	Experimental concentration values [mean ± SD (%Recovery)]			
	T_0_	T_1S_	T_2S_	T_3S_
4 °C	8 ± 1	< MDD	–^a^	–^a^
	49 ± 4	< MDD	–^a^	–^a^
	114 ± 9	13 ± 2 (11%)	–^b^	–^b^

Long-term stability	T_0_	T_1L_	T_2L_	T_3L_
− 20 °C	8 ± 1	9 ± 1 (113%)	8 ± 1 (93%)	11 ± 1 (138%)
	49 ± 4	42 ± 5 (86%)	36 ± 2 (74%)	31 ± 2 (63%)
	114 ± 9	101 ± 5 (89%)	82 ± 5 (72%)	67 ± 5 (59%)
− 80 °C	8 ± 1	8 ± 1 (100%)	10 ± 1 (125%)	11 ± 1 (138%)
	49 ± 4	40 ± 3 (82%)	40 ± 1 (82%)	34 ± 2 (69%)
	114 ± 9	91 ± 7 (80%)	75 ± 6 (66%)	69 ± 6 (61%)

^a^The analysis was not performed because the concentration of NT-proBNP was already lower than MDD at T_1S_.

^b^The analysis was not performed because the concentration of NT-proBNP was already lower than MDD at T_2S_.

### Preliminary clinical assessment

**Figure 2:**
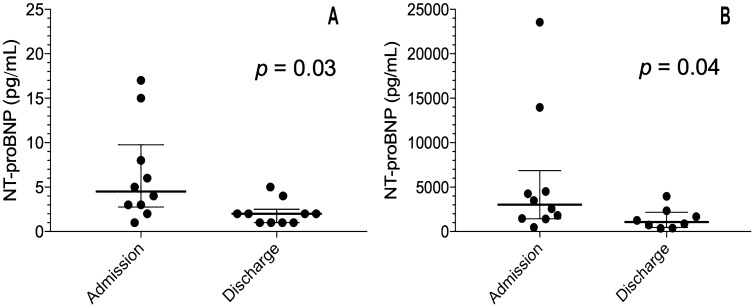
Box-plot for NT-proBNP measured in saliva (**A**) and in blood (**B**). The box-plot shows: the minimum, 25th percentile, median, 75th percentiles, and the maximum value for each variable. Outliers are shown with black points. For both saliva and blood, HF patients at discharge had significantly lower median NT-proBNP values than at admission.

Saliva and blood samples were collected at admission and at discharge from ten patients hospitalized for acute HF at the Fondazione Toscana “Gabriele Monasterio”, Pisa, Italy. On average, a patient was hospitalized for approximately six days. Compared with admission, HF patients at discharge showed significantly lower median (25th and 75th percentile) values of NT-proBNP in blood [3500 pg/mL (1470–10,090 pg/mL) vs. 1200 pg/mL (560–3160 pg/mL), *p* value = 0.04]. Likewise, a significant reduction in the NT-proBNP concentration was observed in saliva [5 pg/mL (2–10 pg/mL) vs. 2 pg/mL (− 3 pg/mL), *p* value = 0.03]. There was an average decrease of about 40% in both saliva and blood. Figure 2 shows the box-plot for NT-proBNP measured in saliva and blood samples.

## Discussion

Blood is one of the best biological fluids to assess systemic processes through the determination of specific biomarkers. Blood NT-proBNP is the gold standard biomarker for HF diagnosis and monitoring, and high levels of NT-proBNP in blood have been associated with cardiac (e.g. ejection fraction), renal (e.g. serum creatinine), and laboratory parameters (e.g. serum potassium and hemoglobin)^44,45^, as well as a higher NYHA class^46^.

Our analytical approach is based on a sandwich enzyme immunoassay kit (the Biomedica Immunoassay) as a proof-of-concept method to obtain useful information on HF by monitoring NT-proBNP in saliva. In 2012, Foo et al.^47^ evaluated an immunochemical assay for NT-proBNP quantification in saliva (NT-proBNP AlphaLISA kit, Perkin Elmer). Foo et al. used the commercial Roche assay to quantify NT-proBNP in plasma samples and to compare analyte levels in the two matrices. However, their approach to saliva analysis requires a pre-concentration step (3 kDa Amicon Ultra-0.5 Centrifugal Filter Devices at 14,000×*g* for 20 min) before performing the ELISA kit. This pre-concentration step provided a limit of detection (LoD) close to 16 pg/mL, recovery between 95 and 110% and intra- and inter-assay CVs below 10%.

Unlike Foo et al., our analytical workflow requires no pre-concentration step and obtains a quantitative salivary recovery. We also obtained a higher NT-proBNP recovery (95–110% vs 85%) and a MDD of 1 pg/mL. Given that we do not need any pre-concentration step, fewer consumables (i.e. centrifugal filters) are required and instrumentation costs are lower. Table 2 compares the performance of the different assays referenced in this paper for plasma/serum and saliva analysis.

**Table 2:** Performance of assays used to determine NT-proBNP in plasma/serum and saliva.

Analytical figure of merit	Plasma/serum			Saliva	
	Elecsys NT-proBNP II assay	NT-proBNP AlphaLISA	Biomedica Immunoassay	NT-proBNP AlphaLISA (Foo et al.)	Biomedica Immunoassay (our work)
Concentration range (pg/mL)	1–35,000	3.9–100,000	0–5424	16–800	1–200
MDD/LoD (pg/mL)	10	3.9	25	16	1
Intra-assay variation (CV%)	N.D	4%	3.5%	7.17%	< 10%
Inter-assay variation (CV%)	N.D	8%	5%	4.46%	< 10%
Recovery (%)	N.D	89–97%	99–108% (serum) 93–94% (plasma)	85%	95 − 110%

Since it is not always possible to analyze samples immediately after collection, we also investigated the stability of NT-proBNP in saliva samples at different storage conditions commonly adopted by clinical laboratories. The simplest sample storage condition (i.e. 4 °C) could not be used for preserving NT-proBNP in saliva since a marked decrease in its levels was observed after 1 week of storage, probably due to the presence of microbial or proteolytic activities capable of degrading the peptide^48^. A longer storage time was obtained by storing saliva samples at − 20 °C and − 80 °C; however, we suggest that all measurements should be performed within 1 month. For example, after 1 month, the concentration of a sample stored at − 20 °C/− 80 °C and analyzed after 5 months was about 30% (CV% = 10%), which is lower than that of a sample stored at the same conditions but analyzed after 1 month. Thus, the measurements of the saliva samples should be carried out at a constant storage time in order to minimize the bias due to the time difference between sampling and analysis. At − 20 and − 80 °C, the concentration of NT-proBNP in saliva was not affected by an additional thawing/freezing cycle. However, if saliva samples are used for a multi-parametric analysis, multiple aliquots should be prepared instead of stressing the samples with more than two freeze–thaw cycles.

An improvement in an HF patient’s health status is correlated with a significant decrease over time of the NT-prBNP in blood^45,49–51^. Even with the limited number of patients enrolled, the aim of our preliminary preclinical assessment was not to investigate a correlation between NT-proBNP salivary and blood levels, but rather to evaluate the possibility of monitoring the trend of NT-proBNP levels in saliva as an alternative indicator of disease progression. Interestingly, NT-proBNP levels in saliva showed a similar behavior to those in blood. A good agreement in the NT-proBNP concentration ratio was observed (Fig. 2), with a significant decrease of 30–40% from admission to discharge. The results of our approach therefore highlighted the potential role of saliva analysis for HF assessment through NT-proBNP monitoring, thus paving the way for future applications using dedicated salivary LoC and PoC devices.

## Study limitations

Although NT-proBNP has already been shown to be a gold-standard biomarker for HF monitoring and saliva analysis has proven to be a powerful alternative matrix to blood, prior research studies on NT-proBNP determination in saliva samples are lacking.

The strengths of this study include the use of saliva as an alternative matrix to blood, the study of NT-proBNP stability in saliva stored at different temperatures, as well as a preliminary evaluation of the trend of salivary NT-proBNP as an indicator of disease progression. Our study concerned the first phase of a preclinical assessment in which saliva was collected from HF patients only at admission (HF acute phase) and at hospital discharge. This limited number of samplings was initially necessary to understand if and how a saliva sampling could be incorporated into hospital routine.

However, one of the main limitations of our study is the extremely low number of patients that it was possible to enroll due to the SARS-CoV-2 public health emergency. In addition, data obtained on NT-proBNP in saliva were compared with blood levels only, without taking into account other possible physiological parameters such as obesity or drug therapy.

Nevertheless, although the number of HF patients enrolled was extremely limited (n = 10), these preliminary findings suggest the diagnostic value of salivary NT-proBNP for HF monitoring due to the correlation (*p* < 0.05) between the trends of NT-proBNP levels in both saliva and blood. It is well known how much inter-variability occurs in clinical studies involving the assessment of the clinical relevance of biomarkers. However, even simply considering the data on NT-proBNP values at the patient admission and discharge, we observed a significant difference in line with HF regression.

## Methods

### NT-proBNP standard solutions

Five lyophilized synthetic human NT-proBNP standard solutions supplied within the ELISA kit were reconstituted in 500 µL of ultrapure water (18.2 MΩ/cm type I ultrapure water, Elga PURELAB Classic) to obtain STDs at 0, 85, 340, 1360, 5420 pg/mL. STD solutions were left on an orbital shaker (80 rpm) at room temperature (22 ± 2 °C) for 10 min before use. These standards were then used to prepare QCSs and spike the saliva samples at the target concentration. Quality control samples (QCSs) and spiked PSSs were prepared by spiking samples at different concentrations using NT-proBNP standard solutions.

### Saliva sampling for assay validation and stability study

Saliva samples were collected from twenty nominally healthy volunteers according to the procedure described elsewhere^27^ using the SOS device. They were then pooled to obtain a PSS which was used for the assay validation and stability study. The volunteers were asked to freely roll the swab in their mouths for about 2 min. Saliva was then recovered by centrifugation at 7000 rpm for 5 min at 4 °C.

### Analyte recovery from sampling device

The analyte recovery from the SOS sampling device was evaluated using PSSs spiked with 10, 50, and 100 pg/mL. An aliquot (1 mL) of each PSS was absorbed into three different swabs. The analyte recovery was calculated from the ratio between the average analyte concentration measured (C_m_) in the samples recovered from the swabs and the spiked concentration (C_s_). In addition, an aliquot (1 mL) of blank sample (milli-Q water, 18.2 MΩ/cm at 25 °C) was absorbed into another three different SOSs to evaluate the possible release of contaminants from the swab material.

### Procedure for NT-proBNP quantification in saliva

NT-proBNP was determined in saliva samples using the enzyme immunoassay for the determination of NT-proBNP in human serum/EDTA plasma, supplied by the Biomedica Immunoassay (Cat. No. SK-1204), following the assay procedure provided by the manufacturer. A wash buffer was prepared by diluting (1:20 v/v) the concentrate buffer supplied in the kit with ultrapure water.

The sandwich enzyme immunoassay was as follows. First, an aliquot (50 µL) of STD, saliva, or QSC was pipetted in duplicate into the wells of the microtiter strips, which were pre-coated with polyclonal sheep anti NT-proBNP antibody. Subsequently, 200 µL of conjugate (sheep anti human NT-proBNP-HRPO) were added into the plates. The plate was then covered tightly by an adhesive strip provided within the kit, and incubated for 3 h at room temperature on a horizontal orbital microplate shaker set at 80 rpm for a gentle swirl. The NT-proBNP in the sample bound itself to the pre-coated antibody in the well and formed a sandwich with the conjugate (detection antibody). Each well-plate was then aspirated and washed five times with 300 µL of diluted wash buffer. In the washing step, all nonspecific unbound material was removed. Subsequently, 300 µL of Tetramethylbenzidine (TMB, Substrate) were pipetted into each well, and the plate was gently swirled again on the orbital shaker for 30 min at 80 rpm in a dark lab-made chamber. The change in color of the catalyzed enzyme in the substrate is directly proportional to the amount of NT-proBNP present in saliva. After the addition of 50 µL of 2 N sulphuric acid (Stop solution), the optical density (OD) was immediately determined at 450 nm and 630 nm. The readings at 630 nm were subtracted from the readings at 450 nm to correct for optical imperfections in the plate. A MultiSkan GO microplate (Thermo Scientific) reader was used to measure the OD.

### Assay validation for NT-proBNP quantification in saliva

Analytical figures of merit such as linearity, matrix effect, sensitivity, recovery, intra- and inter-assay precision were investigated to assess the performance characteristics of the ELISA kit for the analysis of NT-proBNP in human saliva. The validation was performed using STDs solutions supplied in the kit and QCSs prepared by spiking aliquots of PSSs with a known amount of analyte. The blank was also subtracted from the measurements. A PSS was freshly prepared every day of the analysis, which was then used for the assay validation.

Linearity of the assay was evaluated in triplicate in three different ELISA kits by analyzing both STDs and QCSs at six concentration levels of NT-proBNP, in the range of 1–200 pg/mL. The matrix effect was evaluated by comparing, at a confidence level of 95%, the slopes^28, 30^ (reported with the corresponding standard deviation) of the calibration curves obtained from STDs and QCSs in the same concentration range.

The minimum detectable dose (MDD) was determined by adding two standard deviations to the mean optical density value obtained for twenty replicates of unspiked PSS. The corresponding concentration at this level was calculated using the dedicated calibration curve.

QCSs containing 5, 10, 50, 100 and 150 pg/mL of NT-proBNP were used to assess both analyte recovery and assay precision. Analyte recovery was evaluated by comparing the NT-proBNP concentration determined on ten replicates for each QCS with the expected value. Assay precision was evaluated by analyzing each QCS ten times with the same kit for intra-assay precision, and ten times with ten different kits for inter-assay precision.

### Sample stability study

Short- and long-term stability of NT-proBNP in saliva was evaluated at three concentration levels for up to 3 weeks at 4 °C and up to 5 months at both − 20 and − 80 °C, respectively. For this purpose, a PSS obtained at T_0_ was divided into three main aliquots labelled as PSS short-term, PSS_long-term_20, and PSS_long-term_80. Each main aliquot was further divided into three aliquots that were spiked at different analyte concentrations: 10 pg/mL (CL_1_), 50 pg/mL (CL_2_), and 100 pg/mL (CL_3_). All samples were prepared by weighting. . An initial set of samples was analyzed immediately after the collection (T_0_) to obtain the reference values. The remaining samples of CL_1_, CL_2_ and CL_3_ from the PSS short-term were split into three aliquots and then stored at 4 °C. On the other hand, each corresponding sample from PSS_long-term_20 and PSS_long-term_80 were sub-aliquoted into four samples (three aliquots to perform the stability study over time, and one aliquot to investigate the freeze/thaw stability), split into three aliquots and then stored at − 20 and − 80 °C, respectively.

A short-term (T_S_) and a long-term (T_L_) stability study were carried out to evaluate NT-proBNP stability in saliva samples over time. The T_S_ study investigated analyte stability at 4 °C for up to 3 weeks (T_0_: collection day; T_1S_: T_0_ + 1 week; T_2S_: T_0_ + 2 weeks; T_3S_: T_0_ + 3 weeks), whereas the T_L_ investigated analyte stability at − 20 and − 80 °C for up to 5 months (T_0_: collection day; T_1L_: T_0_ + 1 month; T_2L_: T_0_ + 2.5 months; T_3L_: T_0_ + 5 months).

Aliquots of PSS collected and prepared at T_0_ were spiked with 10, 50, and 100 pg/mL of analyte (namely CL_1_, CL_2_, and CL_3_ samples) and immediately analyzed for use as reference values for both T_S_ and T_L_ studies. The effect of two thaw/freezing cycles was evaluated at T_1L_.

**Figure 3:**
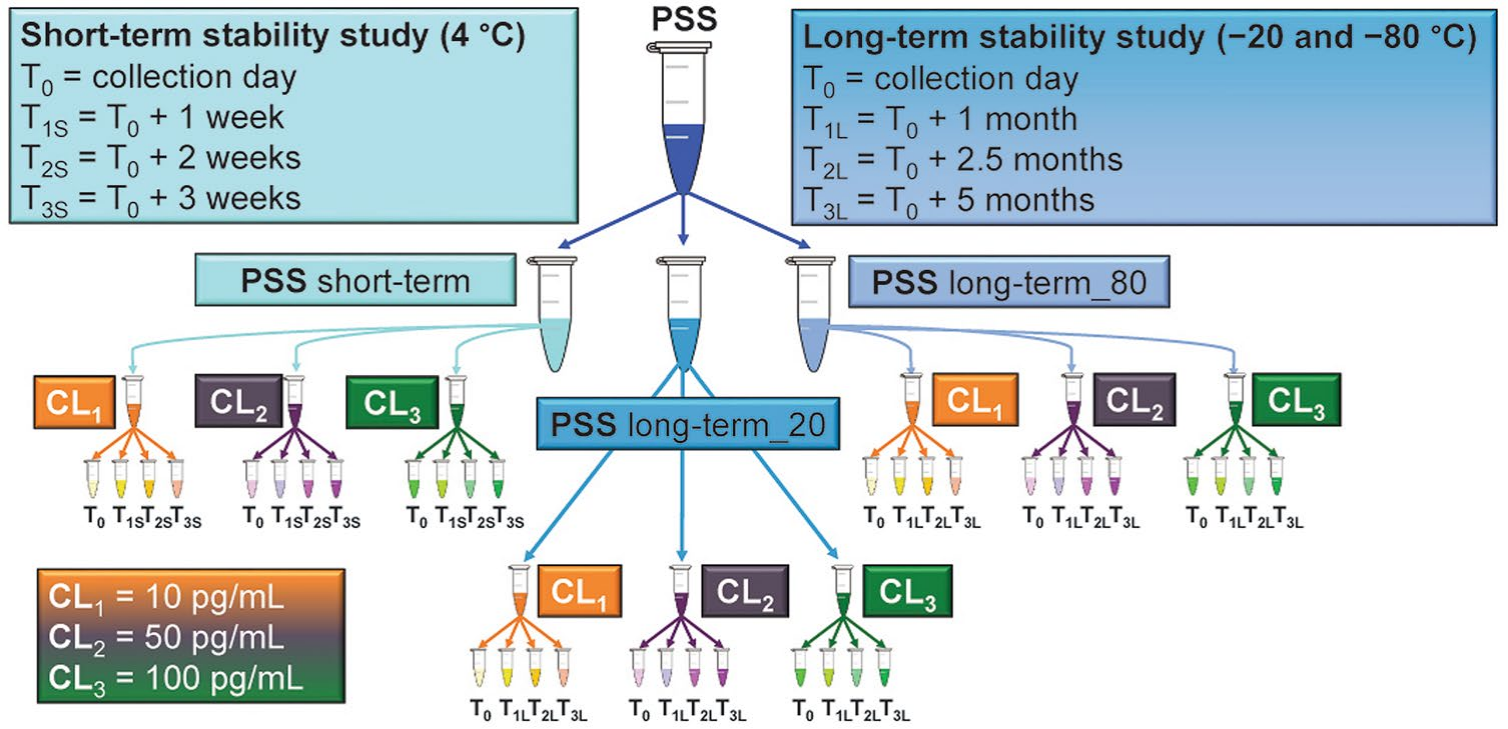
The structure of the stability studies carried out by storing saliva samples containing three different NT-proBNP concentrations (10, 50, and 100 pg/mL) at different temperatures (4, − 20, and − 80 °C). The short-term stability study investigated sample stability stored at 4 °C for up to 3 weeks, whereas the long-time stability investigated sample storage at two temperatures (− 20 and − 80 °C) for up to 5 months. Samples intended for freeze–thawing effect investigations are not included.

Figure 3 shows the experimental plan for the stability study. For the sake of simplicity, samples intended for investigating the freeze–thawing effect are not included.

### Clinical protocol

The study was carried out within the framework of the H2020 KardiaTool project (An integrated PoC solution for diagnosis and therapy monitoring of heart failure patients, Grant No. 768686) and received the approval (protocol number: 54764) of the Ethics Committee of the Area Vasta Nord-Ovest (CEAVNO-Tuscany Region, Italy). The study was conducted in accordance with the 1964 Declaration of Helsinki and subsequent updates. Patient enrolment was carried out from December 2019 to February 2020. The population (7 males, 3 females; age 69 ± 10 years, min age 55 years, max age 89; n = 2 classified NYHA class 2, n = 7 classified NYHA class 3, n = 1 classified NYHA class 4) consisted of patients hospitalized at the Fondazione Toscana Gabriele Monasterio of Pisa, Italy. Informed consent was obtained from all participants included in the study. Blood sample collection was performed at patient admission (T_A_) and at discharge (T_D_) when performing routine clinical analyses, as suggested by the ESC guidelines^10^. Plasma samples were analysed using the hospital procedure based on a commercial automated electrochemiluminescent immunoassay (Elecsys NT-proBNP II, Roche Diagnostics).

Whole saliva was collected at T_A_ and T_D_ between 8 a.m. and 10 a.m. Each subject was asked to refrain from oral hygiene, smoking, eating and drinking for at least 1 h prior to saliva collection. Each subject was also asked to drink water in order to rinse the mouth three times for at least one minute each time. After ten minutes, saliva was collected using a SalivaBio Oral Swab (SOS) (Salimetrics, cod: 5001.02 and 5001.05) according to the following procedure: (1) remove the SOS from package and place it in the subject’s mouth, (2) ask the subject to roll the swab in the mouth for two minutes to collect saliva, avoiding chewing the swab, and (3) remove SOS from the mouth and place it into the container. Samples were kept at − 20 °C. Once the sample was available for analysis, the SOS container was thawed at room temperature and then subjected to centrifuge (7000 rpm, 4 °C, 5 min) to recover saliva. Saliva was then aliquoted (300 µL each) using a micropipette in 1.5 mL Eppendorf LoBind centrifuge tubes.

### Statistical analysis

The normally distributed variables were reported as mean ± standard deviation, whereas skewed variables were described by median with lower (25th percentile) and upper (75th percentile) quartiles. The difference between groups was assessed using a non-parametric test (signed-rank Wilcoxon test). A two-tailed *p* value of < 0.05 was considered statistically significant. The slopes of the calibration curves were compared with the statistical test described by Zar^50^ at a confidence level of 95%. All data were analysed using GraphPad Prism v. 8.0 (GraphPad Software Inc., La Jolla, USA).
